# Recent Advances in the Diagnosis of Talaromycosis

**DOI:** 10.1093/cid/ciaf253

**Published:** 2025-06-26

**Authors:** Lottie Brown, Ngo Thi Hoa, Vo Trieu Ly, Linghua Li, Cunwei Cao, Sirida Youngchim, Methee Chayakulkeeree, Tihana Bicanic, Jasper Fuk-Woo Chan, Kwok-Yung Yuen, Thuy Le

**Affiliations:** Institute of Infection and Immunity, School of Health and Medical Sciences, City St George's, University of London, London, United Kingdom; St George's University Hospitals NHS Foundation Trust, London, United Kingdom; Tropical Medicine Research Center for Talaromycosis, Biomedical Research Center, Pham Ngoc Thach University of Medicine, Ho Chi Minh City, Vietnam; Oxford University Clinical Research Unit, Ho Chi Minh City, Vietnam; Centre for Tropical Medicine, Nuffield Department of Medicine, University of Oxford, Oxford, United Kingdom; Tropical Medicine Research Center for Talaromycosis, Biomedical Research Center, Pham Ngoc Thach University of Medicine, Ho Chi Minh City, Vietnam; Hospital for Tropical Diseases, Ho Chi Minh City, Vietnam; Guangzhou Institute of Clinical Infectious Diseases, Infectious Disease Center, Guangzhou Eighth People's Hospital, Guangzhou Medical University, Guangzhou, China; Department of Dermatology and Venereology, First Affiliated Hospital of Guangxi Medical University, Nanning, Guangxi, China; Department of Microbiology, Faculty of Medicine, Chiang Mai University, Chiang Mai, Thailand; Department of Medicine, Faculty of Medicine, Siriraj Hospital, Mahidol University, Bangkok, Thailand; Institute of Infection and Immunity, School of Health and Medical Sciences, City St George's, University of London, London, United Kingdom; St George's University Hospitals NHS Foundation Trust, London, United Kingdom; State Key Laboratory of Emerging Infectious Diseases, Carol Yu Center for Infection, Department of Microbiology, School of Clinical Medicine, Li Ka Shing Faculty of Medicine, The University of Hong Kong, Pokfulam, Hong Kong Special Administrative Region, China; State Key Laboratory of Emerging Infectious Diseases, Carol Yu Center for Infection, Department of Microbiology, School of Clinical Medicine, Li Ka Shing Faculty of Medicine, The University of Hong Kong, Pokfulam, Hong Kong Special Administrative Region, China; Tropical Medicine Research Center for Talaromycosis, Biomedical Research Center, Pham Ngoc Thach University of Medicine, Ho Chi Minh City, Vietnam; Division of Infectious Diseases and International Health, Duke University School of Medicine, Durham, North Carolina, USA

**Keywords:** talaromycosis, penicilliosis, *Talaromyces marneffei*, *Penicillium marneffei*, diagnosis

## Abstract

Talaromycosis is an invasive fungal disease endemic to Southeast Asia. While culture is essential in identification, susceptibility testing, and typing, the low sensitivity and long turnaround times limit its clinical utility. Several promising monoclonal antibody–based (MAb) antigen-detection assays have been evaluated in large patient cohorts. This includes the MAb-Mp1p and MAb-4D1 enzyme immunoassays and their point-of-care platforms. Quantitative polymerase chain reaction (qPCR) assays targeting the 5.8S or 18S region of ribosomal RNA have been developed. These antigen and qPCR assays are highly specific and more sensitive than blood culture, making them excellent rapid rule-in tests for talaromycosis in people with a compatible clinical syndrome. Metagenomic next-generation sequencing is emerging as a promising tool for non-bias detection of talaromycosis. Host-based diagnostics targeting antibodies, interferon-gamma release, and transcriptomics are being actively developed. This review summarizes recent advances in the diagnosis of talaromycosis and provides expert recommendations on the application of these novel tests to improve the diagnostic algorithm and management of talaromycosis.

## Teaser

This review summarizes recent advances in the diagnosis of talaromycosis and provides expert recommendations on the application of these novel tests alongside conventional microscopy and culture to improve the diagnostic algorithm and management of talaromycosis.

## BACKGROUND

*Talaromyces marneffei* (formerly *Penicillium marneffei*; see Supplementary Appendix 1 for nomenclature change) is a thermally dimorphic fungus that causes a severe, invasive tropical fungal disease known as talaromycosis [1]. Talaromycosis is acquired through inhalation of *T. marneffei* spores from the environment, and can develop as an acute or a latent infection that reactivates up to 50 years after initial exposure [1]. Talaromycosis primarily affects immunocompromised people but occasionally causes disease in immunocompetent hosts. *T. marneffei* is endemic to tropical and subtropical regions of Asia, and is hyperendemic in Vietnam, Thailand, and China, where it has emerged as a leading cause of human immunodeficiency virus (HIV)–related death [2, 3]. The incidence of talaromycosis is rising due to an increase in individuals with other immunocompromising and chronic medical conditions, population migration, and travel [4, 5]. Despite the high mortality rate of up to 30%, talaromycosis receives little investment in research and development, prompting a global call in 2021 for talaromycosis to be recognized as a neglected tropical disease (NTD) [4] and, in 2022, the inclusion of *T. marneffei* on the World Health Organization (WHO) Fungal Priority Pathogen List [6].

## OVERVIEW OF THE CHALLENGES IN THE DIAGNOSIS OF TALAROMYCOSIS

Prompt diagnosis and treatment are critical to preventing deaths from talaromycosis [2]. Clinical features are non-specific and overlap with other opportunistic infections, such as tuberculosis, histoplasmosis, cryptococcosis, and pneumocystosis. A definitive diagnosis relies on isolation of *T. marneffei* in culture from blood or other clinical specimens, which requires 4 to 28 days for results, misses 50% to 70% of infections, and detects disease in its advanced stage when treatment is the least effective [2 ].

## CLINICAL FEATURES ARE BROAD AND NONSPECIFIC

### Patients Without HIV

Talaromycosis can involve many organ systems, and clinical features vary according to the host and level of immunosuppression [7]. In patients without HIV, upper and lower respiratory tract involvement is the most frequent form of infection, including oropharyngeal and bronchial lesions, lung nodules, cavity lung lesions, and pleural effusions [8]. There are an increasing number of reports of primary pulmonary infection in immunocompetent individuals, especially among those with underlying lung diseases such as chronic obstructive pulmonary disease, indicating that *T. marneffei* may be an underrecognized cause of respiratory infection in endemic regions [8]. Extrapulmonary infections of the gastrointestinal tract, urogenital tract, bones, and joints are more common in individuals without HIV [1]. Anti–interferon-gamma (anti-IFN-γ) autoantibody-associated immunodeficiency, which is predominantly found in Southeast Asian people, is the most common underlying risk factor for talaromycosis in apparently healthy individuals, and hence should be tested for [9]. Other non–HIV-associated immunodeficiency conditions due to immune-modulating therapy, malignancy, and inborn errors of immunity are associated with more severe talaromycosis and higher mortality, likely due to delays in recognition and diagnosis [7, 10].

### Patients With HIV

**Figure 1.**
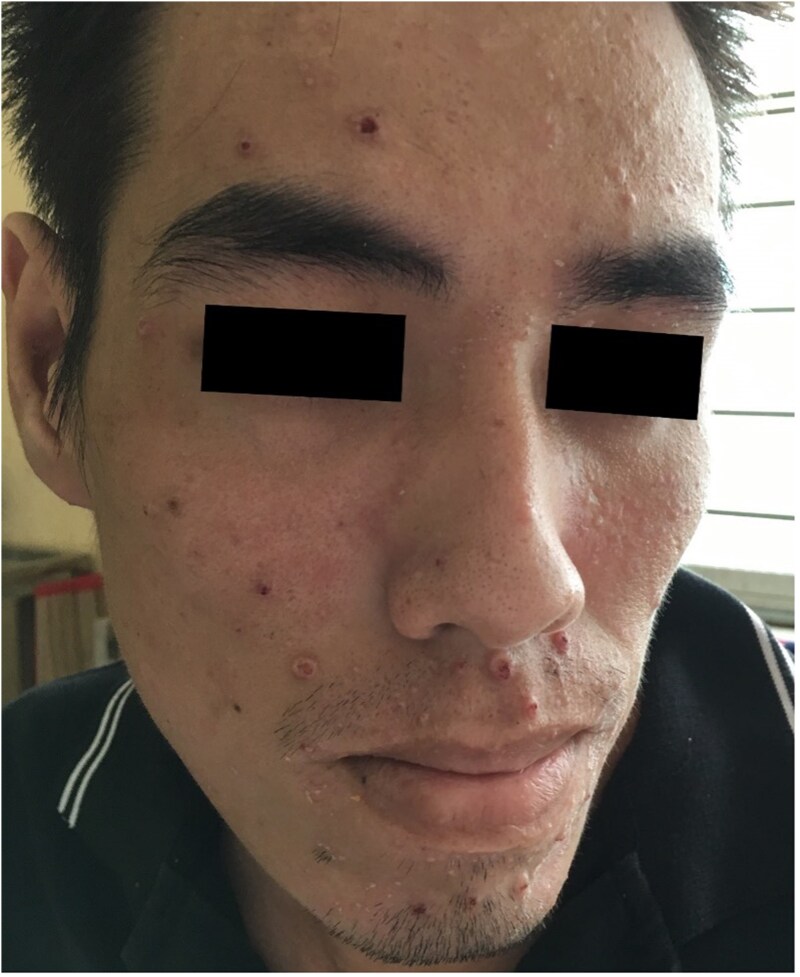
Typical skin lesions on the face of a patient with advanced HIV disease and disseminated talaromycosis in Vietnam. Typical skin lesions are papules with central necrosis that are painless and usually begin on the face and spread to the neck, trunk, and extremities. Abbreviation: HIV, human immunodeficiency virus.

Patients with advanced HIV (CD4 count <100 cells/mm^3^) typically present with disseminated infection and present sub-acutely (median duration of illness of 4 [range: 1–24] weeks). Symptoms and signs are non-specific, including fever, weight loss, fatigue, malaise, gastrointestinal disturbance, hepatosplenomegaly, and lymphadenopathy, for which there is a broad range of possible differential diagnoses [11]. Skin lesions are the most specific sign of disseminated talaromycosis but are late manifestations of talaromycosis and are absent in up to 50% of patients, and even less common in those without HIV (see Figure 1 for typical skin lesions) [12–14]. Common laboratory abnormalities include anemia, thrombocytopenia, and elevated transaminase levels [12]. Concurrent infections with other opportunistic pathogens are common and are associated with poorer prognosis [3].

### Conventional Laboratory Methods for the Detection of *T. marneffei* Microscopy

**Figure 2.**
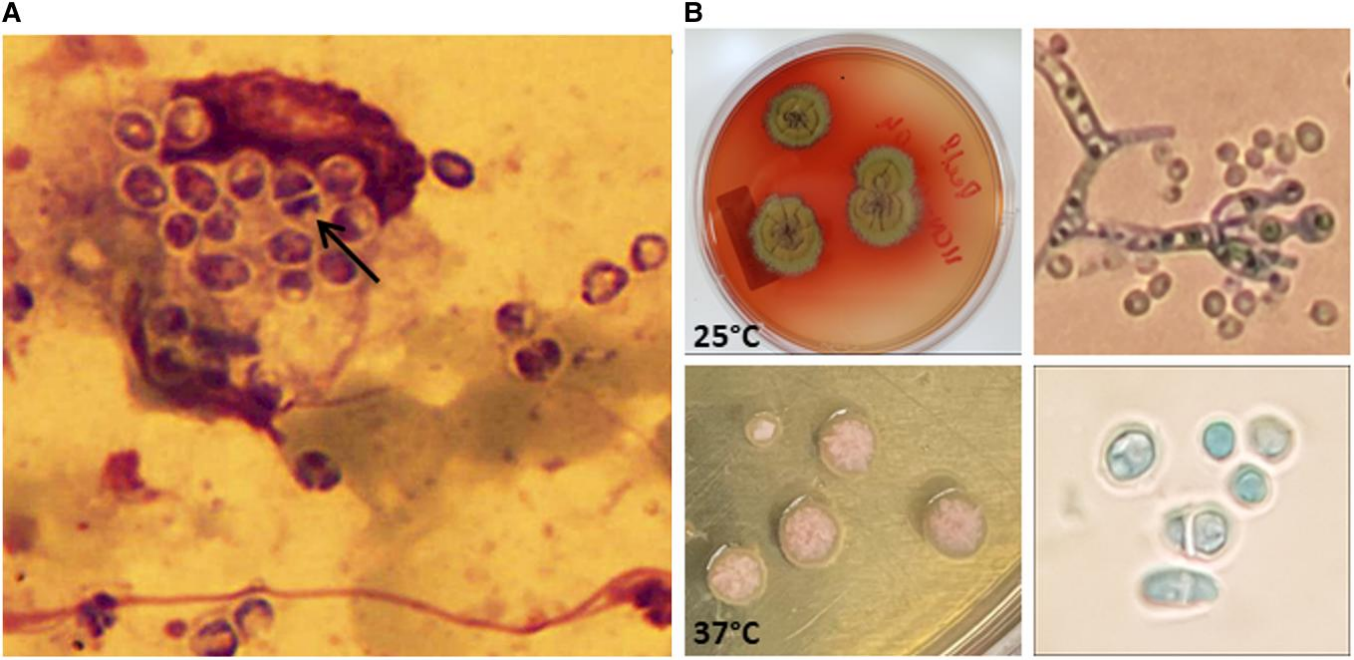
Microscopic and macroscopic appearance of *Talaromyces marneffei. A*, Giemsa stain of the skin smear showing multiple extracellular round-to-oval yeast cells of approximately 3 to 6 µm in size. Many of these are actively dividing with a visible midline septum (indicated by the arrow). *B*, *Talaromyces marneffei* isolated from culture of the patient’s skin lesions. At 25°C, *T. marneffei* produces powdery greenish-yellow mold colonies and a bright red pigment that diffuses into the Sabouraud dextrose agar medium. Tape preparation of the mold colonies shows septate hyphae with conidiophores bearing phialides and round conidia under magnification. At 37°C, *T. marneffei* produces white yeast colonies without the red pigmentation. Microscopic examination shows transitional sausage-like yeast cells, one with a central septum. Other *Talaromyces* species have been reported to cause infections in humans, albeit rarely, but *T. marneffei* can be readily differentiated from other *Talaromyces* as the only *Talaromyces* spp. that exhibits thermal dimorphism, switching from an environmental mold to yeast form at body temperature (≥35°C), and is among the few *Talaromyces* spp. that produce a bright red pigmentation in the mold form.

Microscopic examination of *T. marneffei* in clinical specimens may be performed with Giemsa, Grocott methenamine silver (GMS), Wright staining, or optical brighteners like Calcofluor white to reveal round or oval intracellular or extracellular yeasts 2–3 μm in diameter with a central septation (Figure 2*A*) [1]. A rapid, presumptive diagnosis can be made following visualization of the characteristic central septation of *T. marneffei* yeast cells by microscopy of skin lesion scraping, lymph node aspirate, and tissue biopsy. Peripheral blood microscopy is occasionally positive in cases of high fungemia [1]. Immunofluorescent and immunohistochemical stains may provide improved sensitivity compared with conventional stains but are understudied for *T. marneffei* [15]. Both the sensitivity and specificity of direct specimen microscopy are highly dependent on operator skill and experience. False-negative findings may occur in specimens with low fungal burdens, while *T. marneffei* yeasts may be mistaken for *Histoplasma* and *Emergomyces,* leading to false-positive findings.

### Culture

A definitive diagnosis of talaromycosis relies on isolation of *T. marneffei* in culture from blood, skin lesions, bone marrow, lymph nodes, sputum, or bronchoalveolar lavage fluid. *T. marneffei* is classified as a risk group 2 human pathogen (see Supplementary Appendix 2 for safety considerations). Patients without HIV are less likely to have a positive blood culture compared with patients with HIV [13]. *T. marneffei* can grow within 5 days in standard automated aerobic blood culture systems, and can take up to 28 days to grow on selective solid fungal culture media such as yeast peptone dextrose, Sabouraud dextrose agar (SDA) media, or on selective mycobacterial/fungal blood culture systems [16]. Selective mycobacterial/fungal blood culture (incubated over 42 days) has been shown to increase the detection of *T. marneffei* by 33% compared with standard aerobic blood culture alone, and can detect *Histoplasma* spp., *Mycobacterium tuberculosis*, and non-tuberculous mycobacteria, which are common differential diagnoses of patients suspected to have invasive fungal diseases but that would otherwise be missed by standard blood culture [17 ]. The advantage of the selective mycobacterial/fungal culture bottles is that they can be used in the same machine used for standard aerobic blood culture, so no additional infrastructure is needed. For fungal cultures of non-blood specimens, standard solid culture in an SDA tube or on a plate over 42 days is recommended.

For the identification of positive fungal cultures, the following methods can be used: (1) sub-culturing at 25°C and 37°C to demonstrate dimorphism (which generally takes another 3 days) (Figure 2*B*), (2) matrix-assisted laser desorption/ionization–time-of-flight mass spectrometry (MALDI-TOF MS), and (3) polymerase chain reaction (PCR) and sequencing of the fungal universal Internal Transcribed Spacer (ITS) region. Antifungal susceptibility testing may be performed on positive cultures (see Supplementary Appendix 3 for a summary of minimum inhibitory concentration data).

### MALDI-TOF MS

MALDI-TOF MS equipment requires initial investment but is low cost to run and has high accuracy in identification of *Talaromyces* to the species level, but utility has been limited to laboratories with extensive reference collections [18]. Recent publicly available mass spectral profiles of a comprehensive collection of *T. marneffei* isolates have been developed [18], and efforts to validate these libraries and make them commercially available are ongoing (see Supplementary Appendix 4).

### Antigen Diagnosis

#### Non-specific Fungal Antigen Tests

The *Aspergillus* galactomannan assay has been evaluated for the diagnosis of talaromycosis, with sensitivity ranging between 81% and 96%, although specificity is limited by significant cross-reactivity with *Aspergillus* spp*., Fusarium* spp., and other endemic fungi [19, 20]. Despite the cross-reactivity, invasive aspergillosis or fusariosis is extremely uncommon in patients with advanced HIV disease, so there may be utility in galactomannan in blood as a biomarker of talaromycosis in hyperendemic regions where *T. marneffei*–specific antigen tests are not yet available. Another fungal antigen, serum β-D-glucan (BDG), was elevated in 9 of 11 patients with talaromycosis in a study from Japan [21]; however, further studies are needed to determine its clinical utility. Cross-reactivity with *T. marneffei* has been observed in the *Histoplasma* and *Blastomyces* antigen tests [22, 23].

#### *T. marneffei*–Specific Antigen Tests

**Figure 3.**
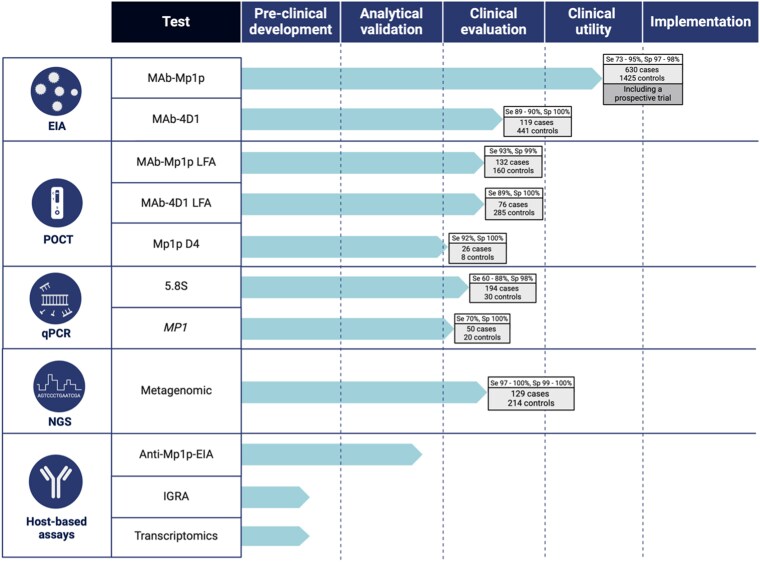
Stages of development of non-culture diagnostics for talaromycosis. Abbreviations: EIA, enzyme immunoassay; IGRA, interferon-gamma release assay; LFA, lateral flow assay; MAb, monoclonal antibody; NGS, next-generation sequencing; POCT, point-of-care test; qPCR, quantitative polymerase chain reaction; Se, sensitivity; Sp, specificity.

Significant advances in antigen testing for talaromycosis have been made in recent years, with the development of 2 promising *T. marneffei*–specific monoclonal antibody–based antigen-detection enzyme immunoassays (EIAs) and their point-of-care platforms (Figure 3). Mp1p antigenemia has been shown to precede blood culture positivity by up to 16 weeks [24], and hence, has the potential to be used for targeted screening of high-risk patients before the onset of clinical symptoms (see Supplementary Appendix 5).

#### Enzyme Immunoassays

##### The 4D1 EIA

The 4D1 EIA uses monoclonal antibodies that target a nonspecified antigen in the whole-cell yeast extract of *T. marneffei.* The 4D1 EIA has excellent analytical specificity, demonstrating no cross-reactivity to a range of clinically related pathogens, including *Histoplasma capsulatum*, *Pneumocystis jirovecii*, *Cryptococcus neoformans*, and *Penicillium* spp [25]. The 4D1 EIA has high clinical sensitivity (89%–100%) and specificity (100%) in urine and serum samples from patients with advanced HIV disease diagnosed with talaromycosis and a wide range of other opportunistic infections (Table 1). However the evaluation of the 4D1 EIA has been limited to retrospective studies using small numbers of blood culture–positive talaromycosis cases—hence, sensitivity may be inflated. Further evaluation including blood culture–positive and blood culture–negative cases is needed to improve its performance estimate.

**Table 1.** Summary of Studies Evaluating Monoclonal Antibody (MAb)–Based Antigen Detection Enzyme Immunoassays (EIAs), Lateral Flow Assays (LFAs), and Other Point-of-Care Assays for Detection of *Talaromyces marneffei* in Clinical Specimens

Assay	First Author (year)	Country	Study Design	Specimens	Se (%)	Sp (%)	Reference
**4D1 EIA**	Prakit (2016)	Thailand	Retrospective cohort of patients with AHD and culture-confirmed talaromycosis (n = 45) and controls without talaromycosis (n = 232), including patients with other invasive fungal diseases (n = 44), bacterial infections (n = 44), HIV and no fungal infections (n = 31), and healthy controls (n = 113)	Serum	100	100	[26]
	Pruksaphon (2021)	Thailand	Retrospective cohort of patients with AHD and culture-proven talaromycosis (n = 76) and without talaromycosis (n = 265), including healthy controls (n = 86) and patients with other infections (n = 184)	Urine	89	100	[27]
	Shu (2023)	Thailand	Retrospective cohort of blood culture–proven talaromycosis with AHD and without HIV (n = 74); controls (n = 229), consisted of healthy individuals (n = 45) and individuals diagnosed with other infections (n = 184)	Urine	89	99	[25]
**Mp1p EIA**	Thu (2020)	Southern Vietnam	Retrospective cohort of patients with AHD and culture-proven talaromycosis (n = 372) and without talaromycosis (n = 517), including healthy volunteers (n = 338) and other infections (n = 179)	Plasma, urine	86	98	[28]
	Chen (2022)	China	Retrospective cohort of patients with AHD and culture-proven talaromycosis (n = 93) and without talaromycosis (n = 190), including other opportunistic infections	Serum	72	97	[29]
	Ly (2023)	Southern Vietnam	Prospective cohort of patients with AHD followed over 6 months (n = 533); a total of 70 developed culture-proven talaromycosis and 463 did not develop talaromycosis	Plasma, serum, urine	89	97	[30]
	Gong (2023)	China	Retrospective cohort of patients with AHD and suspected talaromycosis with fungal culture performed (n = 350), including 95 culture-confirmed cases and 255 controls where there was no evidence of talaromycosis	Serum	72	98	[31]
	Thu (2023)	Northern and southern Vietnam	Multicenter prospective study using Mp1p EIA as a screening tool for talaromycosis patients with AHD, including culture-proven talaromycosis (n = 111), and without talaromycosis (n = 551)	Serum, urine	84	96	[32]
**Mp1p LFA**	Venugopalan (2023)	Southern Vietnam	Retrospective cohort of patients with AHD and culture-proven talaromycosis (n = 132) and without talaromycosis (n = 160)	Plasma, serum, urine	93	99	[33]
**Mp1p D4 POCT**	Kinnamon (2023)	Southern Vietnam	Retrospective cohort of patients with AHD and culture-proven talaromycosis (n = 26) and without talaromycosis (n = 8)	Serum and urine	92	100	[34]

Abbreviations: AHD, advanced HIV disease; D4 POCT; D4 point-of-care test; HIV, human immunodeficiency virus; Se, sensitivity; Sp, specificity.

##### The Mp1p EIA

The Mp1p EIA uses monoclonal antibodies that target a *T. marneffei*–specific fungal cell wall galactomannan Mp1p. The Mp1p is abundantly secreted during infection, and is an important virulence factor for *T. marneffei,* making it an ideal and specific target for immunodiagnostics [35, 36]. The Mp1p EIA has excellent analytical specificity, demonstrating no cross-reaction to several pathogenic fungi, including *H capsulatum*, *P jirovecii*, and *C neoformans* [37]. The Mp1p EIA has been studied more extensively than the 4D1 EIA, and has been evaluated in large retrospective and prospective cohorts of patients with advanced HIV disease at risk of talaromycosis in Vietnam and China (Table 1) [28–32]. Of note, the clinical sensitivity is consistently higher in southern Vietnam (86%–89%) than in northern Vietnam or China (72%–77%) [29, 31]. As the genetic clades of *T. marneffei* in southern and northern Vietnam are distinct [38], this suggests that the Mp1p EIA performance may be *T. marneffei-*clade–specific and requires further investigation. Sensitivity in urine is at least as high as in serum or plasma, and sensitivity is further increased when testing plasma and urine together [28, 30, 32]. The Mp1p EIA has excellent clinical specificity (96% to 100%) when tested in patients with advanced HIV disease with a wide range of opportunistic infections [28, 30, 32]. The clinical sensitivity of the Mp1p EIA is superior to blood culture (84%–86% vs 67%–73%) [28, 30, 32], making it a useful rapid rule-in test. Preliminary results of a multicenter prospective cohort study in Vietnam show a positive-predictive value (PPV) of 77% and a negative-predictive value (NPV) of 97% for the Mp1p EIA as a screening test for talaromycosis in patients with advanced HIV disease, indicating its promising utility not only as a rapid rule-in test but also as a rapid rule-out test for talaromycosis [32].

#### Point-of-Care Antigen Tests

##### The 4D1 Lateral Flow Assay

Both the 4D1 and Mp1p EIAs have been developed into point-of-care lateral flow assays (LFAs) [27, 33]. The 4D1 LFA demonstrated 89% sensitivity and 100% specificity when tested in urine samples from 76 patients with culture-confirmed talaromycosis and 265 healthy controls [27]. Currently, the 4D1 LFA has not been optimized in non-urine samples and has not been evaluated in prospective studies.

##### The Mp1p LFA

The Mp1p LFA was developed by IMMY Diagnostics (Oklahoma, USA). In a case-cohort study of 132 patients with culture-confirmed talaromycosis and 160 controls, the Mp1p LFA had a sensitivity of 93% when testing plasma and urine together and a specificity of 99%, compatible to the performance of the Mp1p EIA performed in the same paired samples [33]. The Mp1p LFA is currently undergoing a multicenter prospective clinical validation in Vietnam [33]. Both the 4D1 and Mp1p LFAs have been developed as qualitative assays, but it is possible to derive semi-quantitative antigen levels using commercially available portable lateral flow strip readers, or by testing serially diluted samples [27]. Lateral flow assays are inexpensive, can be performed near patients without the need for electricity or equipment, and are well suited for point-of-care testing in both hospital and community settings and in both low- and high-resource settings.

##### Mp1p D4 Point-of-Care Antigen Test

The Mp1p D4 point-of-care antigen test is a self-contained, immunoassay platform that utilizes a previously discovered pair of monoclonal and polyclonal antibodies to detect Mp1p antigens [34]. All reagents are integrated within a capillary-driven passive microfluidic cassette that minimizes user intervention and allows the assay to withstand extreme environmental conditions, including high temperatures and high humidity. Antigen quantification can be performed using a portable fluorescence reader. The D4 assay has demonstrated excellent analytical sensitivity, with a limit of detection lower that of the Mp1p LFA (0.2 vs 0.6 ng/mL). However, clinical evaluation of the D4 assay is limited to only 26 cases and 8 controls (92% sensitivity, 100% specificity) [34], with prospective clinical validation ongoing.

### PCR-Based Diagnosis

Several quantitative PCR (qPCR) assays have been developed for the detection of *T. marneffei*, targeting the 5.8S or 18S region of ribosomal RNA (rRNA) or the *MP1* gene encoding Mp1p. Although highly specific (100%), these assays have demonstrated variable sensitivity of between 60% and 86% when tested in various specimens, including in serum, plasma, and whole blood, and clinical evaluation has been limited to retrospective analyses of small numbers of patients (Table 2) [20, 39–42]. In blood culture–positive cases of talaromycosis, the sensitivity of qPCR approaches 100%. In blood culture–negative cases, PCR can detect 55% to 69% of patients in whom a diagnosis requires culture of other clinical specimens, often obtained by invasive biopsy procedures (Table 2) [20, 39–42]. Similar to antigen tests, qPCR assays offer high specificity (close to 100%) and faster (6 hours) turnaround time and reduce the need for invasive biopsy [20, 41].

**Table 2.** Summary of Studies Evaluating qPCR Assays for Detection of *Talaromyces marneffei* on Clinical Specimens

Assay	First Author (year)	Country	Study Cohort	Specimens	Se (%)	Sp (%)	Reference
Nested PCR targeting 18S rRNA	Pongpom (2009)	Thailand	Retrospective cohort of patients with AHD and culture-proven talaromycosis (n = 35)	Serum (n = 35)	67	NE	[40]
qPCR targeting 5.8S rRNA	Pornprasert (2009)	Thailand	Retrospective cohort of patients with AHD with culture-proven talaromycosis (n = 20)	Whole blood (n = 20)	60	NE	[39]
qPCR targeting 5.8S rRNA	Li (2020)	China	Retrospective cohort of patients with AHD and culture-proven talaromycosis (n = 36), including blood culture–positive cases (n = 20) and blood culture–negative cases (n = 16)	Serum (n = 36)	86	NE	[20]
qPCR targeting 5.8S rRNA	Dang Hoang Khanh (2023)	Vietnam	Retrospective cohort of patients with AHD and culture-proven talaromycosis (n = 138) and controls with other opportunistic infections (n = 30)	Whole blood (n = 168)	88	97	[43]
(1**)** Nested PCR targeting ITS region of rRNA; (2) qPCR targeting ITS region of rRNA	Lu (2015)	China	Retrospective cohort of patients with culture-proven talaromycosis (n = 20)	Whole blood (n = 27), serum (n = 3)	(1) 67 (2) 77	NE	[42]
qPCR targeting *MP1*	Hien (2016)	Vietnam	Retrospective cohort of patients with AHD and culture-proven talaromycosis (n = 50) or other opportunistic infections (n = 20)	Plasma (n = 70)	70	100	[41]

Abbreviations: AHD, advanced HIV disease; HIV, human immunodeficiency virus; ITS, Internal Transcribed Spacer; NE, not evaluated; PCR, polymerase chain reaction; qPCR, quantitative polymerase chain reaction; rRNA, ribosomal RNA; Se, sensitivity; Sp, specificity.

The sensitivity of PCR-based methods is highly dependent on an adequate quantity of DNA being released during cell lysis and obtained during the extraction step, particularly in blood culture–negative cases, where the fungal load in the blood is very low. Specific techniques to improve diagnostic sensitivity of qPCR assays are discussed in Supplementary Appendix 6. In a recent evaluation of a 5.8S qPCR assay, DNA extraction using the MasterPure Yeast DNA Purification kit with bead beating on whole blood demonstrated the highest analytical sensitivity to date of 1 yeast cell/mL and high clinical sensitivity of 88% [43 ]. There was no amplification when testing the 5.8S qPCR assay on a range of clinically relevant fungi, including 6 non–*marneffei Penicillium* spp.; 3 *Aspergillus* spp. including *A fumigatus*, *A niger*, and *A terreus*; 4 *Candida* spp.; *H capsulatum*; and *C neoformans* [43]. Similarly excellent analytical specificity is reported with the *MP1* and 18S qPCR assays [40, 41]. Further work is needed to optimize and standardize DNA extraction and PCR amplification protocols for *T. marneffei*. The utility of PCR for prognostication and treatment monitoring is an area of research need.

### Metagenomic Next-Generation Sequencing

Metagenomic next-generation sequencing (mNGS) is a pathogen agnostic approach with the simultaneous detection of all bacteria, viruses, fungi, and parasites present in a clinical specimen. Given the broad and nonspecific clinical presentations of patients, wide range of differentials, and high rates of concurrent infections, mNGS could be an invaluable tool for the diagnosis of talaromycosis. Results for mNGS are typically available in 1–2 days, which is significantly faster than culture in microbiology laboratories with high-throughput sequencing and bioinformatic capability. Investigations into the use of mNGS for talaromycosis are limited to case reports and 3 small case series, which have shown excellent diagnostic performance for diagnosing talaromycosis on a variety of specimen types and have confirmed a high rate of concurrent infections (Table 3) [44–46]. In comparison to blood culture, sensitivity and specificity of mNGS approached 100% with positivity rates of mNGS higher than culture, which could indicate superior sensitivity but may also represent false-positive findings. Universal standards to interpret mNGS results and define infection based on the number of reads are needed. In specimens with very low fungal burdens (such as blood culture–negative cases of talaromycosis), the sensitivity of mNGS may be lower than qPCR, due to the lack of amplification step. Finally, the requirement for expensive equipment, extensive infrastructure, and logistical and technical support limits its use in low-resource settings where *T. marneffei* is endemic.

**Table 3.** Summary of Studies Evaluating mNGS for Detection of *Talaromyces marneffei* on Clinical Specimens

Platform	First Author (year)	Country	Study Cohort	Specimens	Se (%)	Sp (%)	Reference
MGISEQ-2000 or MGISEQ-50	Mao (2022)	China	Retrospective cohort of patients with AHD and suspected opportunistic infections (n = 208) in China, with culture-confirmed talaromycosis (n = 60) and without talaromycosis (n = 148)	BALF	98	99	[44]
Illumina Nextseq CN500	Zhang (2024)	China	Retrospective cohort of patients with AHD and without HIV with other immunocompromising diseases, including culture-confirmed talaromycosis (n = 40) and suspected talaromycosis but negative by culture (n = 38)	Mixed (blood, tissue, BALF, sputum)	100	NE	[45]
Illumina NextSeq	Jiang (2024)	China	Retrospective cohort of patients without HIV diagnosed with other immunocompromising diseases; cases were patients with culture-confirmed talaromycosis (n = 29) and controls were diagnosed with other infections (n = 28)	Mixed (BALF, skin, lymph nodes, blood, and others)	97	100	[46]

Abbreviations: AHD, advanced HIV disease; BALF, bronchioalveolar lavage fluid; HIV, human immunodeficiency virus; NE, not evaluated; Se, sensitivity; Sp, specificity.

### Host-Based Assays

The application of pathogen-based assays is limited to patients with clinically apparent or advanced stage of talaromycosis. Host-based diagnostics have the potential to detect latent or clinically silent infection, facilitating understanding of the clinical epidemiology of talaromycosis (ie, past exposure to *T. marneffei*), akin to assays in screening for latent tuberculosis. Host-based assays may have a role in screening for latent infection in high-risk individuals, such as individuals undergoing impending immunosuppressive therapy, chemotherapy, or organ and bone marrow transplantation, for antifungal prophylaxis to prevent talaromycosis reactivation. An anti-Mp1p immunoglobulin G (IgG) antibody detection EIA has been developed [36]. A recent study found that, although the sensitivity is low (only 103/315 patients with HIV-associated talaromycosis had positive anti-Mp1p IgG antibodies), IgG response was associated with a 9-fold higher chance of survival compared with no IgG response, suggesting an important role of host humoral response in disease pathogenesis [47]. A host-based IFN-γ release assay (IGRA) for diagnosing latent talaromycosis is under development [48]. Potential diagnostic biomarkers are being identified through transcriptomic analysis, such as components of the sphingolipid signaling pathway, which is activated in macrophages infected with *T. marneffei* [49].

## CONCLUSIONS AND RECOMMENDED APPROACH TO TALAROMYCOSIS DIAGNOSIS

The current diagnosis of talaromycosis relies on conventional microscopy and culture, which lack sensitivity, miss approximately half of all infections, and only detect *T. marneffei* in the late stages of infection when treatment is less likely to be effective. The Mp1p EIAs and their point-of-care platforms have shown excellent clinical utility in making an early diagnosis, and have promising utility in predicting disease development and in monitoring of treatment response. Quantitative PCR tests have demonstrated high specificity and superior sensitivity compared with blood culture, and thus have potential as rapid rule-in tests for talaromycosis. There is room for further optimization of DNA extraction and PCR procedures to further improve assay sensitivity, especially among blood culture–negative patients.

**Figure 4.**
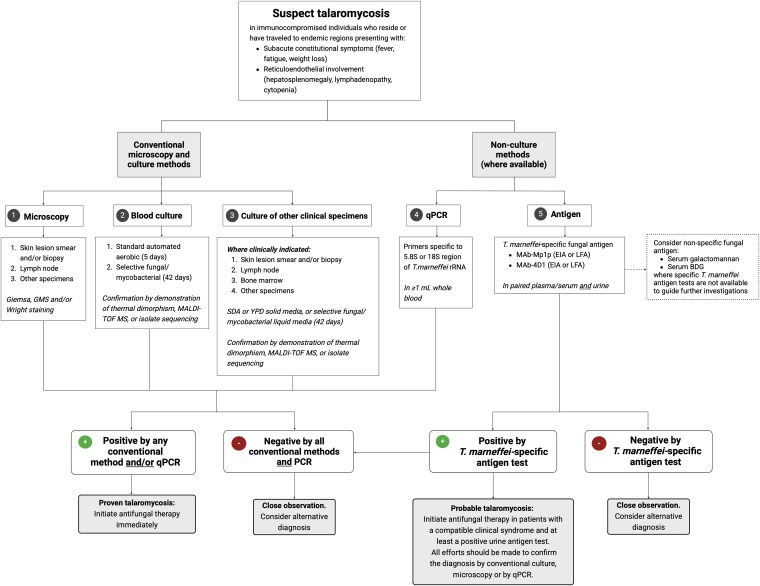
Diagnostic and management algorithm for talaromycosis. Despite the many advantages of antigen and qPCR assays, they should not replace conventional microscopy and culture of blood, skin lesions, lymph nodes, and other specimens. Conventional methods remain critical gold-standard tests and should be performed in all patients with suspected talaromycosis as they add to the total diagnostic yield in non-blood clinical specimens and allow for antifungal susceptibility testing in patients failing treatment. Specific fungal/mycobacterial culture should be implemented where available. qPCR positivity is highly specific for talaromycosis, and can serve as a rapid rule-in test. Positive antigen results should be considered true positives only in symptomatic patients with clinically compatible symptoms. While a positive galactomannan or BDG may be indicative of talaromycosis in patients at risk, all efforts should be made to confirm infection by conventional culture methods or specific PCR-based detection techniques. A negative galactomannan or BDG result does not rule out infection. Abbreviations: BDG, β-D-glucan; EIA, enzyme immunoassay; GMS, Grocott methenamine silver; LFA, lateral flow assay; MAb, monoclonal antibody; MALDI-TOF MS, matrix-assisted laser desorption/ionization–time-of-flight mass spectrometry; PCR, polymerase chain reaction; qPCR, quantitative polymerase chain reaction; rRNA; ribosomal RNA; SDA, Sabouraud dextrose agar; YPD, yeast peptone dextrose.

Figure 4 shows our recommended diagnostic and management algorithm for talaromycosis, incorporating antigen and qPCR testing into the conventional microscopy and culture diagnostic and management algorithm. Positive qPCR results from blood and sterile clinical specimens should be considered true positives and require treatment. Positive antigen results should be considered true positives only in symptomatic patients with clinically compatible symptoms. Selective fungal/mycobacterial blood culture should be implemented where available, as this will improve the diagnostic yield in low-fungal-burden patients during early stages of disease, and will also pick up histoplasmosis, tuberculosis, and non-tuberculous mycobacteria, which are highly prevalent in patients susceptible to talaromycosis.

## Supplementary Material

ciaf253_Supplementary_Data

## Supplementary Data

Supplementary materials are available at *Clinical Infectious Diseases* online. Consisting of data provided by the authors to benefit the reader, the posted materials are not copyedited and are the sole responsibility of the authors, so questions or comments should be addressed to the corresponding author.

## Notes

***Author contributions.*** T. L. conceived the review. L. B. wrote the first draft of the manuscript and produced the medical illustrations in collaboration with T. L. and N. T. H. All authors contributed to the writing of the manuscript. All authors have agreed to the final manuscript and have final responsibility for the decision to submit for publication.

***Financial support.*** L. B. is funded by the National Institute for Health Research (NIHR) and received fellowship grants from the Federation of European Microbiological Societies, Dowager Countess Eleanor Peel Trust, the British Society for Medical Mycology, and the Wingate Foundation. T. L. receives research funding from the National Institutes of Allergy and Infectious Diseases (grant numbers R01 AI181764-01, R01AI177098-02, R01-AI143409-05, U01AI169358-03).

***Data availability***. Data in this systematic review were extracted from published and preprint studies available on the internet.
