# Supplementary material for: Recent Advances in the Diagnosis of Talaromycosis
Source: Clin Infect Dis. 2025 Jun 26;81(6):e533–43. doi: 10.1093/cid/ciaf253 (PMC13375561; doi:10.1093/cid/ciaf253)
Supplement: ciaf253_Supplementary_Data [file ciaf253_supplementary_data.docx]

**Supplementary Appendix: Recent Advances in the Diagnosis of Talaromycosis**

Lottie Brown MSc^1,2^, Ngo Thi Hoa PhD^3,4,5^, Vo Trieu Ly MD^3,6^, Linghua Li MD^7^, Cunwei Cao PhD^8^, Sirida Youngchim PhD^9^, Methee Chayakulkeeree PhD^10^, Tihana Bicanic PhD^1,2^, Jasper Fuk-Woo Chan MD^11^, Kwok-Yung Yuen MD^11^, Thuy Le PhD^3,12^

**Affiliations**

1. *Institute of Infection and Immunity, School of Health & Medical Sciences, City St George’s, University of London, London, UK*
2. *St George’s University Hospitals NHS Foundation Trust, London, UK*
3. *Tropical Medicine Research Center for Talaromycosis, Biomedical Research Center, Pham Ngoc Thach University of Medicine, Ho Chi Minh City, Vietnam*
4. *Oxford University Clinical Research Unit, Ho Chi MinH City, Vietnam*
5. *Centre for Tropical Medicine, Nuffield Department of Medicine, University of Oxford, Oxford, UK*
6. *Hospital for Tropical Diseases, Ho Chi Minh City, Vietnam*

*7. Guangzhou Institute of Clinical Infectious Diseases, Infectious Disease Center, Guangzhou Eighth People's Hospital, Guangzhou Medical University, Guangzhou, People's Republic of China.*

*8. Department of Dermatology and Venereology, First Affiliated Hospital of Guangxi Medical University, Nanning, Guangxi, China.*

*9. Department of Microbiology, Faculty of Medicine, Chiang Mai University, Chiang Mai 50200, Thailand.*

*10. Department of Medicine, Faculty of Medicine Siriraj Hospital, Mahidol University, Bangkok, Thailand.*

1. *State Key Laboratory of Emerging Infectious Diseases, Carol Yu Center for Infection, Department of Microbiology, School of Clinical Medicine, Li Ka Shing Faculty of Medicine, The University of Hong Kong, Pokfulam, Hong Kong Special Administrative Region, China*
2. *Division of Infectious Diseases and International Health, Duke University School of Medicine, Durham, NC, USA*

**(1) Recent change in *Talaromyces marneffei* nomenclature**

*T. marneffei* was previously classified under the *Penicillium* subgenus *Biverticillium* based on morphological features. Further phylogenetic research led to the transfer of *Penicillium* subgenus *Biverticillium* to *Talaromyces* and renaming of *Penicillium marneffei* to *T. marneffei* in 2011*^1^.* Other *Talaromyces* species have been reported to cause infections in humans, albeit rarely, including *T. amestolkiae*, *T. purpurogenus* and *T. piceu*s. *T. marneffei* can be readily differentiated from other *Talaromyces* as the only *Talaromyces* spp. that exhibits thermal dimorphism, switching from an environmental mold to yeast form at body temperature (≥ 35°C), and is among the few *Talaromyces* spp. that produce a bright red pigmentation in the mold form^2,3^.

**(2) Safety Considerations for the Laboratory Diagnosis of Talaromycosis**

T. marneffei is classified as a risk group 2 human pathogen; hence all open fungal cultures (even when working with the non-infectious yeast form) should be handled inside a class II Biological Safety Cabinet (BSCII) both in endemic and non-endemic settings, including procedures to extract fungal proteins for MALDI-TOF MS identification.

**(3) Antifungal Susceptibility Testing**

A major advantage of culture is the ability to perform antifungal susceptibility testing using either broth dilution methods or E-test. Like all dimorphic fungi, antifungal susceptibility of *T. marneffei* varies between the mold and yeast form^4^. The reported minimum inhibitory concentrations (MICs) among various studies and among various antifungal susceptibility testing methods are generally consistent. *T.* *marneffei* is highly susceptible to the azole drugs itraconazole, voriconazole, posaconazole, isavuconazole (0.001 µg/ml to 0.07 µg/ml)^4-7^, and 5-fluorocytosine (5-FC) (0.015 µg/ml to 1.0 µg/ml)^7-9^. MICs are intermediate for amphotericin B (0.12 µg/ml to 4.0 µg/ml)^7^ but clinically amphotericin B is very potent against talaromycosis^10^. MICs are higher for fluconazole (0.25 µg/ml to 16.0 µg/ml)^8^ and the echinocandins (2 to 8 µg/ml), suggesting that they are unlikely to be effective^5,6,8,9^. Studies of new antifungal drugs are limited but olorofim fosmanogepix, and oteseconazole have shown low MICs^9^. The MIC cut-off to define clinical activity of antifungal drugs against *T. marneffei* have not been established. However, in a recent study examining 101 cases of talaromycosis, cases of slow fungal clearance, defined as persistence of blood culture positivity after 2 weeks of antifungal therapy with amphotericin B, exhibited higher MICs for both voriconazole and fluconazole (*χ^2^* = 12.623, *P* < 0.001 and *χ^2^* = 9.356, *P* = 0.002, respectively), suggesting the possibility of drug resistance^11^.

**(4) Matrix-Assisted Laser Desorption/Ionization-Time of Flight Mass Spectrometry Commercial Databases**

*T. marneffei* identification has recently been added to the Bruker Biotyper system but still requires database expansion with local strains in endemic regions^12^. *T. marneffei* is not yet included in the Vitek MS IVD Database (Biomerieux, Marcy l’Etoile, France)^13^. In a recent evaluation of a new MALDI-TOF MS system, the Zybio EXS3000 (Zybio Inc., Chongqing, China) correctly identified 100% of the 135 *T. marneffei* strains from four distinct clusters^8^.

**(5) The role of antigen testing for screening, prognostication, and monitoring of treatment response**

Mp1p antigenemia detected by EIA has been shown to precede blood culture positivity by up to 16 weeks^14,15^, and therefore has the potential to be used for targeted screening of high-risk patients with advanced HIV disease before the onset of clinical symptoms^16^. This may facilitate disease prevention through a screen-and-treat strategy with the potential to reduce mortality^17^. Quantification of antigen levels is possible with the Mp1p EIA using a standard curve of known concentrations of the recombinant Mp1p, or of the Mp1p LFA using a lateral flow strip reader^18-20^. The ability to quantify antigen levels presents an opportunity to improve clinical management, in predicting disease severity, monitoring treatment response, and evaluating treatment effect of novel antifungal strategies.

**(6) Approaches to Increase the Diagnostic Sensitivity of *T. marneffei* PCR**

Whole blood as a specimen is shown to outperform plasma in sensitivity as it allows detection of both intracellular and extracellular fungal DNA. *T. marneffei* replicates inside macrophages, which may be lost during serum or plasma specimen processing^21^. As demonstrated for other fungal pathogens like *Aspergillus* and *Mucorales*, DNA extraction of high volumes of whole blood (≥ 1 – 3 mL), elution of DNA in smaller volumes (< 50 μl) and reducing the ratio of DNA extract to the final volume of PCR reaction may improve diagnostic yield, particularly in patients with negative blood culture^22-26^. The use of bead-beating prior to DNA extraction, to mechanically facilitate the release of fungal DNA from yeast cells, improves the recovery of fungal DNA, and requires only 1-2 minutes additional time with minimal equipment. For the extraction of *Histoplasma* spp. DNA, bead-beating has been shown to increase the yield 100-fold^26^. In a recent evaluation of a 5.8S qPCR assay, DNA extraction using the MasterPure Yeast DNA Purification kit with bead beating on whole blood demonstrated the highest analytical sensitivity to date of 1 yeast cell ml^-1^ and high clinical sensitivity of 88%^21^. The addition of bead beating prior to DNA extraction by the MasterPure Yeast DNA Purification kit resulted in improved assay performance compared to sonication or to no bead beating (*P* = 0.0064).”

**Supplementary References**

1. Tsang C-C, Tang JYM, Lau SKP, Woo PCY. Taxonomy and evolution of Aspergillus, Penicillium and Talaromyces in the omics era – Past, present and future. *Computational and Structural Biotechnology Journal* 2018; **16**: 197-210.

2. Li L, Chen K, Dhungana N, Jang Y, Chaturvedi V, Desmond E. Characterization of clinical isolates of Talaromyces marneffei and related species, California, USA. *Emerging Infectious Diseases* 2019; **25**(9): 1765.

3. Le T, Houbraken J, Hagen F. Misidentification of Talaromyces marneffei in wild animal and human nasal samples in Portugal - Red pigment production is not unique. *Res Vet Sci* 2023; **164**: 105035.

4. Tan XT, Binti Mohd Shuhairi N, Jane Ginsapu S, Binti Shukor S, Amran F. Comparison of in vitro Susceptibilities of Talaromyces marneffei in Mold and Yeast Forms in Malaysia. *Infect Drug Resist* 2023; **16**: 1629-35.

5. Lau SK, Lo GC, Lam CS, et al. In Vitro Activity of Posaconazole against Talaromyces marneffei by Broth Microdilution and Etest Methods and Comparison to Itraconazole, Voriconazole, and Anidulafungin. *Antimicrob Agents Chemother* 2017; **61**(3).

6. Lei HL, Li LH, Chen WS, et al. Susceptibility profile of echinocandins, azoles and amphotericin B against yeast phase of Talaromyces marneffei isolated from HIV-infected patients in Guangdong, China. *European journal of clinical microbiology & infectious diseases : official publication of the European Society of Clinical Microbiology* 2018; **37**(6): 1099-102.

7. Supparatpinyo K, Nelson KE, Merz WG, et al. Response to antifungal therapy by human immunodeficiency virus-infected patients with disseminated Penicillium marneffei infections and in vitro susceptibilities of isolates from clinical specimens. *Antimicrob Agents Chemother* 1993; **37**(11): 2407-11.

8. Fang L, Liu M, Huang C, et al. MALDI-TOF MS-Based Clustering and Antifungal Susceptibility Tests of Talaromyces marneffei Isolates from Fujian and Guangxi (China). *Infect Drug Resist* 2022; **15**: 3449-57.

9. Zhang J, Liu H, Xi L, Chang YC, Kwon-Chung KJ, Seyedmousavi S. Antifungal susceptibility profiles of olorofim (formerly F901318) and currently available systemic antifungals against mold and yeast phases of Talaromyces marneffei. *Antimicrobial Agents and Chemotherapy* 2021; **65**(6): 10.1128/aac. 00256-21.

10. Le T, Kinh NV, Cuc NTK, et al. A Trial of Itraconazole or Amphotericin B for HIV-Associated Talaromycosis. *The New England journal of medicine* 2017; **376**(24): 2329-40.

11. Guo P, Chen W, Chen S, et al. The delayed clearance of Talaromyces marneffei in blood culture may be associated with higher MIC of voriconazole after antifungal therapy among AIDS patients with talaromycosis. *PLOS Neglected Tropical Diseases* 2023; **17**(4): e0011201.

12. Borman AM, Fraser M, Szekely A, Johnson EM. Rapid and robust identification of clinical isolates of Talaromyces marneffei based on MALDI-TOF mass spectrometry or dimorphism in Galleria mellonella. *Medical Mycology* 2019; **57**(8): 969-75.

13. Lee H, Koo J, Oh J, et al. Clinical Evaluation of VITEK MS PRIME with PICKME Pen for Bacteria and Yeasts, and RUO Database for Filamentous Fungi. *Microorganisms* 2024; **12**(5).

14. Ly VT, Thanh NT, Thu NTM, et al. Occult Talaromyces marneffei infection unveiled by the novel Mp1p antigen detection assay. Open forum infectious diseases; 2020: Oxford University Press US; 2020. p. ofaa502.

15. Pruksaphon K, Intaramat A, Simsiriwong P, et al. An inexpensive point-of-care immunochromatographic test for Talaromyces marneffei infection based on the yeast phase specific monoclonal antibody 4D1 and Galanthus nivalis agglutinin. *PLoS neglected tropical diseases* 2021; **15**(5): e0009058.

16. Longley N, Jarvis JN, Meintjes G, et al. Cryptococcal antigen screening in patients initiating ART in South Africa: a prospective cohort study. *Clinical Infectious Diseases* 2016; **62**(5): 581-7.

17. Ly VT, Nhut VM, Chau NVV, Le T. DIAGNOSTIC MODEL OF TALAROMYCOSIS IN HIV/AIDS PATIENTS. *Tạp chí Y học Việt Nam* 2023; **532**(2).

18. Shu F, Pruksaphon K, Nosanchuk JD, Thammasit P, Youngchim S. Evaluation of the yeast phase-specific monoclonal antibody 4D1 and Galanthus nivalis agglutinin sandwich ELISA to detect Talaromyces marneffei antigen in human urine. *Frontiers in Cellular and Infection Microbiology* 2023; **13**: 1163868.

19. Prakit K, Nosanchuk J, Pruksaphon K, Vanittanakom N, Youngchim S. A novel inhibition ELISA for the detection and monitoring of Penicillium marneffei antigen in human serum. *European Journal of Clinical Microbiology & Infectious Diseases* 2016; **35**: 647-56.

20. Thu NT, Chan JF, Ly VT, et al. Superiority of a novel Mp1p antigen detection enzyme immunoassay compared to standard BACTEC blood culture in the diagnosis of talaromycosis. *Clinical Infectious Diseases* 2021; **73**(2): e330-e6.

21. Dang Hoang Khanh PTHM, Nguyen Thi Mai Thu, Vo Trieu Ly, Nguyen Thanh Hiep, Ngo Thi Hoa, Thuy Le. Development and clinical evaluation of a 5.8S rRNA quantitative PCR assay for the diagnosis of talaromycosis. The National Scientific Conference on HIV/AIDS. Hanoi, Vietnam; 2023.

22. White PL, Alanio A, Brown L, et al. An overview of using fungal DNA for the diagnosis of invasive mycoses. *Expert Review of Molecular Diagnostics* 2022; **22**(2): 169-84.

23. Millon L, Scherer E, Rocchi S, Bellanger A-P. Molecular Strategies to Diagnose Mucormycosis. *Journal of Fungi* 2019; **5**(1): 24.

24. White PL, Bretagne S, Klingspor L, et al. Aspergillus PCR: one step closer to standardization. *J Clin Microbiol* 2010; **48**(4): 1231-40.

25. White PL, Perry MD, Loeffler J, et al. Critical stages of extracting DNA from Aspergillus fumigatus in whole-blood specimens. *J Clin Microbiol* 2010; **48**(10): 3753-5.

26. Alanio A, Gits-Muselli M, Lanternier F, et al. Evaluation of a new Histoplasma spp. quantitative RT-PCR assay. *The Journal of Molecular Diagnostics* 2021; **23**(6): 698-709.

27. Xu H, Nguyen T, Venugopalan S, et al. 622. Development of Interferon-Gamma Release Assays for Diagnosing Latent Talaromycosis: Open Forum Infect Dis. 2023 Nov 27;10(Suppl 2):ofad500.688. doi: 10.1093/ofid/ofad500.688. eCollection 2023 Dec.
